# Functional Ferroic Domain Walls for Nanoelectronics

**DOI:** 10.3390/ma12182927

**Published:** 2019-09-10

**Authors:** Pankaj Sharma, Peggy Schoenherr, Jan Seidel

**Affiliations:** 1 School of Materials Science and Engineering, UNSW Sydney, Sydney, NSW 2052, Australia; 2 ARC Centre of Excellence in Future Low-Energy Electronics Technologies (FLEET), UNSW Sydney, Sydney, NSW 2052, Australia

**Keywords:** ferroelectrics, multiferroics, domain walls, topological defects, nanoelectronics

## Abstract

A prominent challenge towards novel nanoelectronic technologies is to understand and control materials functionalities down to the smallest scale. Topological defects in ordered solid-state (multi-)ferroic materials, e.g., domain walls, are a promising gateway towards alternative sustainable technologies. In this article, we review advances in the field of domain walls in ferroic materials with a focus on ferroelectric and multiferroic systems and recent developments in prototype nanoelectronic devices.

## 1. Introduction

Materials research involving nanoscale properties of functional materials, especially in correlated electron systems, is faced with rich fundamental physics and a wide variety of phenomena that can potentially be exploited for new technology and applications. These systems present a great range of interesting aspects, such as superconductivity, magnetism, topological insulators, and ferroelectricity, to name just a few. Ferroic materials (i.e., materials with some form of built-in order defined by the order parameter), possess a spontaneous polarization, spontaneous strain, and spontaneous magnetization or toroidal order. These materials can change their properties in a preconceived manner through the application of external fields. Thus, given their intrinsic structure, ferroic materials are ideally suited for functionality on the nanoscale. Ferroic phases typically arise in two or more distinct orientations of the order parameter; that is, they can form domains of uniform order which are separated by domain walls.

Domain walls are one type of topological defect that can be found in ordered solid-state materials. The concept of using such topological defects as functional nanoscale elements [1] has given rise to recent developments of prototype domain wall nanoelectronics elements. These include diodes [2], nonvolatile memory [3], and tunnel junctions [4], among others. In all these examples, domain walls take the role of the active device part (e.g., for storing information in memory), and their absence or presence defines the resistance state (high or low) of the element. These element’s functions are driven by external stimuli, such as electric or magnetic fields, stress, or currents. Manipulation by electric fields is especially interesting, as it typically requires less energy than current-driven circuits, which is a key aspect for the development of ultra-low-power electronics technology based on such materials. In addition, the recent demonstration of optical control of domain walls in ferroelectrics [5] points towards the opportunity of developing new optoelectronic domain wall elements that can be controlled and manipulated by light.

In this review, we confine ourselves to the latest advances in the field of domain walls in ferroic materials, with a focus on ferroelectric and multiferroic systems. Multiferroic materials correspond to systems exhibiting several ferroic orders. We start from a discussion of the basic properties of domain walls and later look into recent developments in prototype nanoelectronic devices.

## 2. Intrinsic Structure and Chirality of Domain Walls

The transition of the order parameter in ferroic domain walls has been widely discussed from a theoretical perspective [6,7,8,9]. For multiferroic systems, it was pointed out that order parameter coupling, structure, and symmetry changes at the wall can give rise to new properties [10,11]. Associated electronic properties of walls have been discussed as well [12,13,14,15] in addition to phase transitions [16] or chemical changes within walls [17]. One interesting aspect of recent research is that ferroelectric and multiferroic domain walls that were believed to be predominantly Ising type actually often exhibit mixed character with Bloch, Neel, and Ising components [18]. This finding has sparked many new ideas and developments in basic research and potential application concepts of domain walls. At the forefront of this work are detailed experimental atomic-scale investigations, especially by electron microscopy techniques [19,20] and theoretical advances, which have led to ongoing exploration and exploitation of intrinsic structure of domain walls, such as chirality [21,22,23] Bloch, and Ising lines [24,25]. These features provide interesting and emerging aspects for nanoscale functional domain wall elements [26] and their manipulation (the concept of “a wall within a wall”), which could enable further developments for nanoelectronics. For example, moving kinks (atomic scale “roughness” or steps) in domain walls have been shown to be supersonic, opening possibilities in high-frequency electronics applications [27]. The opportunity of having localized phase transitions within the wall only, such as metal-insulator transitions [28] or transitions from a chiral to a nonchiral state, are very interesting concepts for topological electronics or ‘topotronics’. Besides, investigation of distinct electronic properties, such as electrical conduction at walls in insulating ferroic oxides, and their interconnection with the intrinsic wall structure is yet another aspect that is fundamental to nanoelectronic applications of domain walls and is discussed further in the following section.

## 3. Conduction at Domain Walls

Since initial reports of domain wall conduction in WO_3_ [29] and BiFeO_3_ (BFO) [30,31], the phenomenon has been observed in a range of materials systems such as uniaxial ferroelectrics [28,32,33,34,35] (LiNbO_3_; Pb(Zr/Ti)O_3_; LiTaO_3_; BaTiO_3_, KTiOPO_4_), multiferroics and improper ferroelectrics [36,37,38,39] (*R*MnO_3_, *R*: Er, Y, Dy, Lu; (Ca,Sr)_3_Ti_2_O_7_; Cu_3_B_7_O_13_Cl), and magnetic systems [40,41]. Domain walls in ferroelectrics or multiferroics are characterized by the angle between polarization vectors in neighboring domains and their charge state. Depending on the polarization reorientation across the wall, uncharged (head-to-tail or side-by-side 180° oriented dipoles), positively (head-to-head) and negatively (tail-to-tail) charged walls can be created.

**Figure 1:**
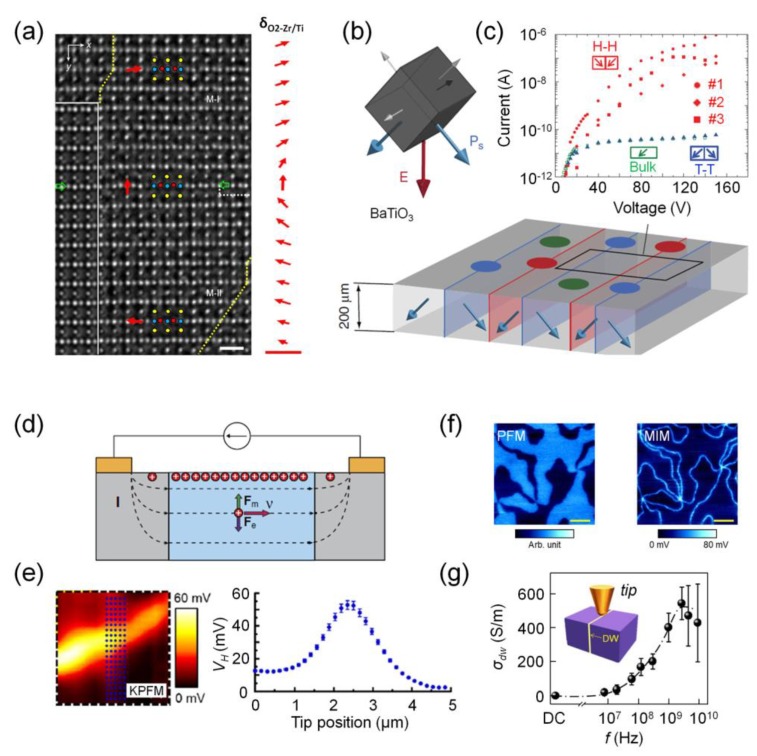
Non-Ising nature of walls (**a**): Atomically resolved transmission electron microscopy (TEM) image of the Neel-like continuous rotation of oxygen displacements across domain wall viewed along the [100]_M_ direction. Green arrows denote domain wall. Scale bar shown in the TEM image is 0.5 nm. From the TEM image, the calculated evolution of the oxygen-displacement vector across the domain wall is shown on the right; scale bar, 30 pm [23]. Quasi 2 DEG (dimensional electron gas) at wall (**b**,**c**): A schematic showing electrical poling of a BaTiO_3_ single crystal in the (110)_c_ direction (red arrow) that allows two equally likely ferroelectric domain states (blue arrows) out of the six permitted (blue and grey arrows) (**b**). With this poling, tail-to-tail (T-T) and head-to-head (H-H) domain walls with periods ranging from 100 to 300 mm are formed in a 200 mm thick (110)_c_ sample. *I-V* characteristics for bulk, H–H, and T-T domain walls (**c**) [28]. Hall measurements (**d**,**e**): Schematic of Hall effect measurements using SPM techniques (**d**) [57]. Spatially resolved map of hall voltage (*V_H_*) and its cross-section profile across a T-T domain wall in an ErMnO_3_ single crystal (**e**) [58]. AC conduction at domain walls (**f**,**g**): Piezoresponse force microscopy (PFM) and sMIM (frequency 1 GHz) images acquired on (001) ErMnO_3_ single crystal (**f**). Domain wall conductivity (σ_DW_) versus frequency data for (001) YMnO_3_ (**g**). Inset in (**g**) shows sMIM tip measuring domain wall [59].

Domain wall conduction can be observed at charged as well as uncharged domain walls. However, uncharged domain walls are much more common, and they are usually energetically favorable and more stable. The general consensus on the observation of electrical conduction at uncharged ferroelectric domain walls is that it arises due to the following factors or combinations: structure-driven concomitant changes in the electronic structure leading to a reduced band-gap in the wall region [30,42]; chiral character of the wall [22,43]; inhomogeneous strains [44]; and accumulation of charged defects at the wall [31,32,45]. Wall regions can be considered as high-symmetry regions reflecting the crystal structure and electronic properties of the high-temperature cubic paraelectric phase of perovskite oxides. The paraelectric phase (e.g., of BFO [46]), is more conducting than its ferroelectric phase, whereas the reverse holds true for the improper multiferroic YMnO_3_ [47]. Consequently, the domain walls in BFO are found to be more conducting [30,31], and in YMnO_3_ more resistive, than the domains [47]. This is validated by density functional theory computations that reveal a reduced bandgap for the cubic paraelectric phase of BFO [42]. In addition, chiral walls promote rigid rotation of polarization across the wall (see Figure 1a). This causes a polarization discontinuity and an electrostatic potential step at the wall. Theoretical modeling supports this scenario and predicts an electrostatic potential step for all wall types in multiferroic BFO [42] and 90° ferroelectric–ferroelastic domain walls in Pb(Zr/Ti)O_3_ [8] and BaTiO_3_ [48]. Even for uniaxial ferroelectrics with 180° domain walls, which were earlier thought to be mainly Ising-like (no potential step), theoretical calculations [18,44,49,50,51,52,53] and recent experimental evidence points to their mixed character that includes both Bloch-like and Neel-like components [23,25]. This mixed character can lead to a potential step at the domain wall. The electrostatic potential step promotes accumulation of screening charges at the wall plane, which presumably leads to its enhanced electrical conduction. Furthermore, theoretical calculations also point to the role of inhomogeneous elastic strains that exist at the wall. Local inhomogeneous strains are predicted to reduce electronic bandgap at multiferroic walls through deformation and flexoelectric coupling (i.e., coupling between strain gradients and polarization) [44,53].

Local measurement methods, especially scanning probe microscopy (SPM), have been successfully used to study nominally uncharged domain wall conduction. Charge transport has been analyzed in the framework of various models ranging from Schottky emission [30,31,54], Poole–Frenkel type [32], space-charge-limited (SCL) transport [31,32], Fowler–Nordheim tunneling [54], and thermally activated charged defect-assisted transport [32,55]. The found heterogeneity of electronic transport along with wide variations in reported domain wall conductivity reflects the importance of defect chemistry, charged defects, and growth conditions. Oxygen vacancies acting as electron donors are one of the most commonly occurring defects in thin-film synthesis of ferroelectric oxides and have been suggested to play a critical role in domain wall conduction. For thin films of BFO, oxygen vacancies are suggested to be responsible for electron dominated or *n*-type conduction at its domain walls [30,31,54]. This, however, is at odds with theoretical investigations that consider BFO to be a *p*-type semiconductor [56] supported by recent experimental results on polycrystalline BFO [55] showing Fe^4+^ and Bi vacancy accumulation at domain walls, suggesting *p*-type hopping conduction. Careful control and understanding of defect chemistry of ferroelectrics are, thus, necessary to critically analyze observed anomalous electrical conduction at walls. Nevertheless, these conducting interfaces are active at the nanometer length scale and can be reconfigured using external electric fields presenting potential opportunities for nanoelectronic devices. To fully harness their potential requires a greater degree of control and understanding of domain wall conduction properties. In particular, commonly seen nominally uncharged domain wall currents of few pA need to be scaled up to make fast and reliable nanoelectronic device operations possible.

Charged domain walls, in that regard, offer a viable option, as much higher wall currents have been predicted [60,61,62,63,64,65] and reported [28,36,37,38,39,66,67,68,69,70] because of a large polarization discontinuity at the wall facilitating an increased accumulation of free charges. Charged domain walls have higher energy of formation and, thus, are usually unfavorable [71]. However, given their ubiquitous observations in various ferroelectrics [28,67,68,72], the formation of stable or metastable charged domain walls is indeed possible, provided there are enough free charge carriers and/or mobile charged defects available for compensation. A quasi-two-dimensional electron gas has been theoretically suggested and observed at 90° head-to-head charged domain walls in BaTiO_3_ (BTO) single crystals that show metal-like conduction ~10^9^ times that of the domains and tail-to-tail walls (Figure 1b,c) [28]. Stable charged domain walls in BTO single crystals were created upon cooling the material through its phase transition under a strong electric bias. In LiNbO_3_ [67], positively charged domain walls were created by active writing current stabilization and were shown to display conductivity (wall currents in the μA range) approximately 13 orders of magnitude larger compared to the bulk. Another approach involving an electrically biased moving SPM tip that generates a directional in-plane electric field, called trailing field [73], has been used to create highly conducting, rewritable, stable charged domain walls [74] and one-dimensional ferroelectric domain vortices [75]. Inducing stable head-to-head domain walls in BFO using this approach leads to currents of a few nA, which are at least 2 to 3 orders of magnitude higher than those seen for uncharged domain walls [74]. However, elastic interactions at boundaries between poled and unpoled regions have been argued to induce instability for trailing field written domains [76].

More recently, exotic crossed quadrant center convergent and divergent domain structures [77,78,79] that allow for head-to-head and tail-to-tail domain wall configurations, respectively, were reported in nanostructures of BFO. These radially convergent and divergent states were electrically addressable, and conductivity at walls was shown to be tunable by three orders of magnitude. Additionally, in improper ferroelectrics, where the polarization is not the primary order parameter, stable charged domain walls can occur naturally and are not necessarily fully screened [80]. In hexagonal manganites (h-*R*MnO_3_, *R* = Sc, Y, In, Dy-Lu), for example, the geometrically driven polarization shows meandering domain walls with different conductivities depending on the wall angle relative to the polar direction [36,37,38,39].

Apart from influencing the charged state of domain walls by the polarization direction in neighboring domains, the electronic properties of ferroelectric domain walls can also be controlled through chemical doping. Bulk conductance of the improper ferroic *p*-type semiconductor ErMnO_3_ was shown to be readily tunable, that is, enhanced and suppressed with substitution of *A*-site aliovalent Ca^2+^ and Zr^4+^ dopants, respectively [81,82]. Specifically, for a Ca content of ~1%, current densities at tail-to-tail charged domain walls increased by more than two orders of magnitude [83]. This study [83] further indicated the possibility of achieving even higher current densities in hexagonal manganite thin films by doping up to 15%, at which the crystalline hexagonal ferroelectric phase of the materials is suppressed. For *B*-site substitution with donor dopant Ti^4+^ at 0.2% doping level, the bulk conduction decreased by an order of magnitude leading to a reduced domain wall conduction [84]. Besides *A*– and *B*– site chemical doping, oxygen vacancies also significantly impact domain wall conduction [31,54,85]. Oxygen vacancies in La-doped BFO thin films were suggested to preferentially accumulate at walls. Temperature-dependent measurements revealed a thermally activated transport process and the role of ionized oxygen vacancies enabling electrical transport with activation energies of ~0.24 to 0.5 eV [54]. Further, in Pb(Zr/Ti)O_3_ (PZT) thin films [85] a transition between conducting and insulating domain wall states was ascribed to oxygen vacancy redistribution that takes place in combination with adsorbates on the surface of the film.

Despite recent advances in understanding electronic properties at ferroelectric domain walls, little is known about their carrier types, charge densities, or mobilities. The intrinsically small size of ferroelectric domain walls together with their occurrence in highly insulating materials linked with difficulties in fabricating Ohmic contacts makes standard measurement protocols, such as Hall measurements, challenging. Recent progress in the detection of the Hall effect was made by customized SPM techniques (e.g., intermittent-contact atomic force microscopy (AFM) [57] and Kelvin probe force microscopy (KPFM) [58]), by driving an electrical current through a wall in the presence of an applied perpendicular magnetic field (Figure 1d,e). In multiferroic YbMnO_3_ single crystals, *p*-type conduction was found at tail-to-tail domain walls with an estimated carrier density of ~10^16^ cm^−3^ and mobility of ~50 cm^2^V^−1^s^−1^, which is about an order of magnitude below equivalent mobilities seen in *p*-type silicon [57]. KPFM experiments revealed carrier densities of ~10^13^ cm^−3^ and much higher mobilities on the order of several 100 cm^2^V^−1^s^−1^ at tail-to-tail domain walls in ErMnO_3_ [58].

These reported room temperature mobilities are relatively large (e.g., compared to at the LaAlO_3_−SrTiO_3_ heterointerface and oxide conductors) and present opportunities for the development of high-speed nanoelectronics. Furthermore, domain walls themselves behave as functional nanodevices with interesting electronic properties that include memristive behavior [86], electronic switches [87], diode-like rectifiers [88], and alternating current conduction at microwave frequencies (Figure 1f,g) [59,89,90,91].

## 4. Light Interaction with Domain Walls

Light-matter interaction is one of the promising fields in physics with already established applications in lasers, optoelectronic devices, and solar energy converters. New developments in light interacting with multiferroic domain walls, especially the electric polarization of walls, can be split into three main categories:

- Light can function as a detection tool to investigate ferroic properties at the walls.
- Light can be used to manipulate domain and domain-wall states.
- Domains and domain walls in ferroelectrics exhibit a photovoltaic (PV) effect much like solar cells.

**Figure 2:**
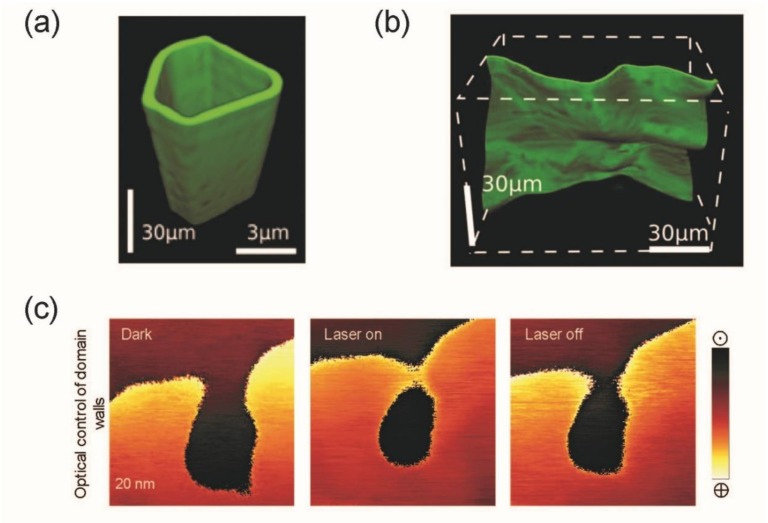
Optical 3D visualization of domain walls (**a**,**b**): Cherenkov second-harmonic generation (SHG) on LNO revealing the three-dimensional shape of 180° side-by-side (**a**) and head-to-head (**b**) domain walls [98]. Optical domain manipulation (**c**): Ferroelectric domain wall motion in KNN-BNNO [(K_0.5_Na_0.5_)NbO_3_]_x_/[Ba(Ni_0.5_Nb_0.5_)O_3−δ_]_1−x_ induced by laser radiation due to photo-induced charging of the surface [103].

Diverse optical tools have been used to resolve ferroelectric domain structures including Raman spectroscopy [92,93] optical coherence tomography [94], nonlinear Talbot imaging [95], defect-luminescence microscopy [96], electro-optical near-field microscopy [97], and different types of second-harmonic generation (SHG) (normal, Cherenkov-type, near-field, confocal) [98,99,100,101]. Especially SHG, which corresponds to a doubling of the incoming light frequency if the system possesses a broken symmetry, is an optimal tool to probe ferroic properties, as all ferroic orders are a direct result of a broken symmetry in the system (e.g., ferroelectricity breaks the inversion symmetry). SHG has, thus, been used to reveal embedded domain and domain wall structures of multiple (multi-)ferroic materials [102]. Even though optical techniques are limited in resolution, SHG-based methods can non-invasively profile the shape of domain walls in all three dimensions. Recently, Cherenkov SHG measurements mapped the three-dimensional shape of charged domain walls in lithium niobate (LNO) [98,100]. As intensively discussed in the previous section, charged domain walls in ferroelectric materials have triggered intensive research over the last few years, but their conductivity has been mostly investigated by surface-sensitive techniques (SPM). However, especially in bulk crystals, the shape of these buried domain walls is crucial for their conductivity. LNO is an extensively studied uniaxial, ferroelectric material that forms 180° domain walls. Cherenkov SHG revealed that side-by-side domain walls are mostly straight (Figure 2a), and their charging can be controlled via their inclination angle in agreement with surface-sensitive piezoresponse force microscopy (PFM) studies [33]. The inclination angle depends on specific electric field poling and annealing procedures. In contrast, fully charged domain walls (head-to-head or tail-to-tail), induced by heat treatment in the vicinity of the Curie temperature, exhibit a more complex domain wall texture distinguishing them from straight side-by-side walls [98] (compare Figure 2a,b).

In addition to SHG, confocal Raman spectroscopy, as well as optical coherence tomography, have been used to image embedded domain boundaries and ferroelectric domains of bulk LNO [92,94,104,105]. Optical techniques can also study polarization reversal processes, as shown in strontium barium niobate by Ayoub et al. [106], or even analyze enhanced conductivity at domain walls (e.g., in LNO domain walls as shown by Godau et al. [69]). Additionally, using light to probe ferroic properties opens the possibility to study time-resolved and dynamical processes [101,107]. For example, optical coherence tomography has shown to exhibit a high temporal resolution to measure real-time domain wall dynamics under changing electric fields [38].

Light is a powerful tool to characterize (multi-)ferroic properties; however, higher fluxes can also be used to influence them. An all-optical approach (i.e., a contact-free process to control polar domain structures and their domain walls) has advantages over standard electric field poling. Most of the electrical poling processes require patterned electrodes, which can limit resolution as well as reduce flexibility in crystal orientation. Almost a decade ago, Warren et al. [108] showed that exposure to light can influence the polarization in a material. Extensive experiments on LNO have presented light-assisted polarization switching, where samples were irradiated with UV light either during or prior to electrical switching [109,110,111,112,113]. The reduction in switching fields can be up to an order of magnitude and can be a consequence of local heating of the irradiated area [112,113].

In LNO, even a full reversal of polarization is possible by pure irradiation [114]. The process was found to be propagated by a heat-assisted diffusion of lithium ions [115,116]. This process was found by Steigerwald et al. [115] to be much more likely than the one proposed first, which was mediated by a heat-induced depolarization field [114], because of an immediate screening of a depolarization field at elevated temperatures preventing a flip in polarization. Another indication for the lithium-ion diffusion model is the polarization reversibility that only works on the +c-face of LNO, which can be explained by the unidirectionality of the thermoelectric effect [117]. A thermoelectric effect could also account for the domain reversibility in strontium barium niobate (SNB) [117]. However, screening charges, increased conductivity at elevated temperatures, photoinduced charges, as well as pyroelectric effects could have an impact on the reversal, and more investigations are needed.

Most of the light-induced polarization reversal work that one finds in the literature is done using UV irradiation. Because of the limited penetration depth of UV light, this only creates surface domains. Experiments performed by Chen et al. [118] showed how this can be overcome. By using tightly focused infrared light pulses, with LNO being highly transparent in the infrared range, poled domain regions can extend several micrometers into the bulk.

An all-optical approach to switch the polarization direction can also be used to flip antiferromagnetic domains. Experiments on multiferroic orthorhombic TbMnO_3_, which has a direct coupling between its magnetic and polar order, have shown full control over the creation and annihilation of ferroelectric domains and, thus, control over its antiferromagnetic domains [119].

Other ways to control the polarization by light in ferroelectric materials include the photovoltaic effect. The bulk photovoltaic effect (BPV) in ferroelectric materials has been already known for many years, with renewed interest due to increasing demand for renewable energies. The photovoltaic effect in ferroelectrics is induced by broken inversion symmetry, which leads to different scattering, relaxation, or photo-excitation probabilities depending on the propagation direction of charge carriers. This can lead to efficient charge separation and, thus, to a photovoltaic effect. Review papers on the BPV in ferroelectrics can be found elsewhere [120,121,122]. The discovery of an abnormal photovoltaic effect, corresponding to an open circuit voltage far above the bandgap, led to an increased interest in ferroelectric materials. The part that domain walls play in the photovoltaic process has been highly controversial. Yang et al. [123,124] ascribed the high photovoltages to electrostatic potential steps at ferroelectric domain walls leading to efficient separation of charge carriers. Each potential step at a ferroelectric domain wall leads to an efficient charge separation similar to a *p–n* junction. In a strip domain pattern, the voltage would add up at each domain wall leading to an open circuit voltage far above the bandgap. Their findings on BFO 71° domain walls support this model by showing that the total PV voltage linearly increases with more domain walls/potential steps between two electrodes. This voltage build-up is only possible if the domain walls run parallel to the electrodes. Measurements with domain walls perpendicular to the electrodes showed, thus, no open-circuit voltages. They also state that the BVP in their experiments is negligible because of a high recombination rate. Several reports agree on the step potential model, but their experiments also exhibit a BPV effect [125,126,127]. For example, measurements on multidomain BTO gave large positive photocurrents compared to a negative BPV detected in a single domain structure. Their results could only be explained by electrostatic potential steps at the domain walls in combination with a BPV effect in the domains [126]. Recent simulations also confirmed the existence of an electrostatic potential step at 71° and 109° domain walls in BFO [128].

In contrast, Alexe and co-workers found no enhanced PV effect at ferroelectric domain walls in local SPM-based PV measurements [129,130]. Their experiment suggested that the abnormal PV effect is solely induced by the bulk, where subbands inside the bandgap could play an important role in inducing asymmetry in k-space that enables high open-circuit voltages [131]. Additionally, the enhanced photocurrent that they measured at the domain walls was assigned to a higher conductivity rather than a PV effect [132]. A higher conductivity at the walls could even be detrimental for the PV effect, as they can act as shunts between electrodes [133].

So far, it has been only established that ferroelectric domain walls play an important role in the PV effect, but their exact mechanism is still under debate. However, because of their impact, the optical properties of ferroelectric materials can be influenced by controlling the domain pattern. Yang et al. showed that by switching the net in-plane polarization by 180° in BFO the photo-induced voltage changes its polarity [127]. Such approaches could give a much higher local flexibility than in conventional semiconductor-based PV devices.

The photovoltaic effect can also be used to manipulate the ferroelectric state. Vats et al. [103] ascribed this effect to be the reason for their domain wall movement and domain growth in KNN-BNNO [(K_0.5_Na_0.5_)NbO_3_]_x_/[Ba(Ni_0.5_Nb_0.5_)O_3−δ_]_1−x_ (see Figure 2c). By exposure of light, photo-excited charges accumulate at the surface and induce an electric field. If the electrical field overcomes the coercive field, it influences the polarization. Rubio-Marcos and co-workers have taken a different photo-driven approach. They influenced the domain wall motion by rotating the light polarization (parallel to the surface). In their experiments, they showed that in BTO [134], as well as KNN [72], a photostrictive effect occurs in the material influencing the domain wall motion as well as domain growth. A photostrictive effect can be seen as a superposition of a photovoltaic and inverse piezoelectric effect. They found that their charged 90° domain walls interacted the most with the polarization direction of the incoming light, leading to strain changes that induced a reversible domain wall motion.

Overall, the experiments on light-domain wall (ferroelectric) interactions show a vast and interesting field of phenomena. Most of all they show that there is still a lot to be discovered and explained, which could lead to new prospects in future applications.

## 5. Magnetic Properties

So far, we have mainly discussed ferroelectric properties at (multi-)ferroic domain walls. In this section, we want to look at the magnetic properties in combination with electronic components in domain walls. Linking both orders can lead to new ways of manipulating electric and magnetic characteristics of multiferroic materials. The coexistence and coupling of magnetic and ferroelectric order are determined by the magnetoelectric effect. Depending on whether both ferroic orders appear independently or jointly, one talks about a type I or type II multiferroic, respectively. Extensive reviews on different types of magnetoelectric multiferroics can be found elsewhere [135,136]. Here, we will concentrate on both orders appearing at the domain wall.

Magnetoelectric domain walls can occur in type I multiferroics, even though both ferroic orders appear independently. Experiments have shown that both multiferroic, as well as purely ferroic domain walls, exist in these materials. Extensive optical and SPM studies on hexagonal manganites (h-RMnO_3_) revealed their highly complex domain structures. In h-RMnO_3_, each ferroelectric domain wall is accompanied by an antiferromagnetic wall with additional free antiferromagnetic domain walls [137,138,139]. In another type I multiferroic, BFO, which is a very rare case of a single-phase magnetoelectric multiferroic at room temperature, 71° and 109° ferroelectric domain walls are accompanied by a change in magnetic structure [9]. Studying domain patterns in type I multiferroics and understanding the magnetic/electric coupling opens the possibility of controlling the ferroelectric component by a magnetic field and vice versa.

**Figure 3:**
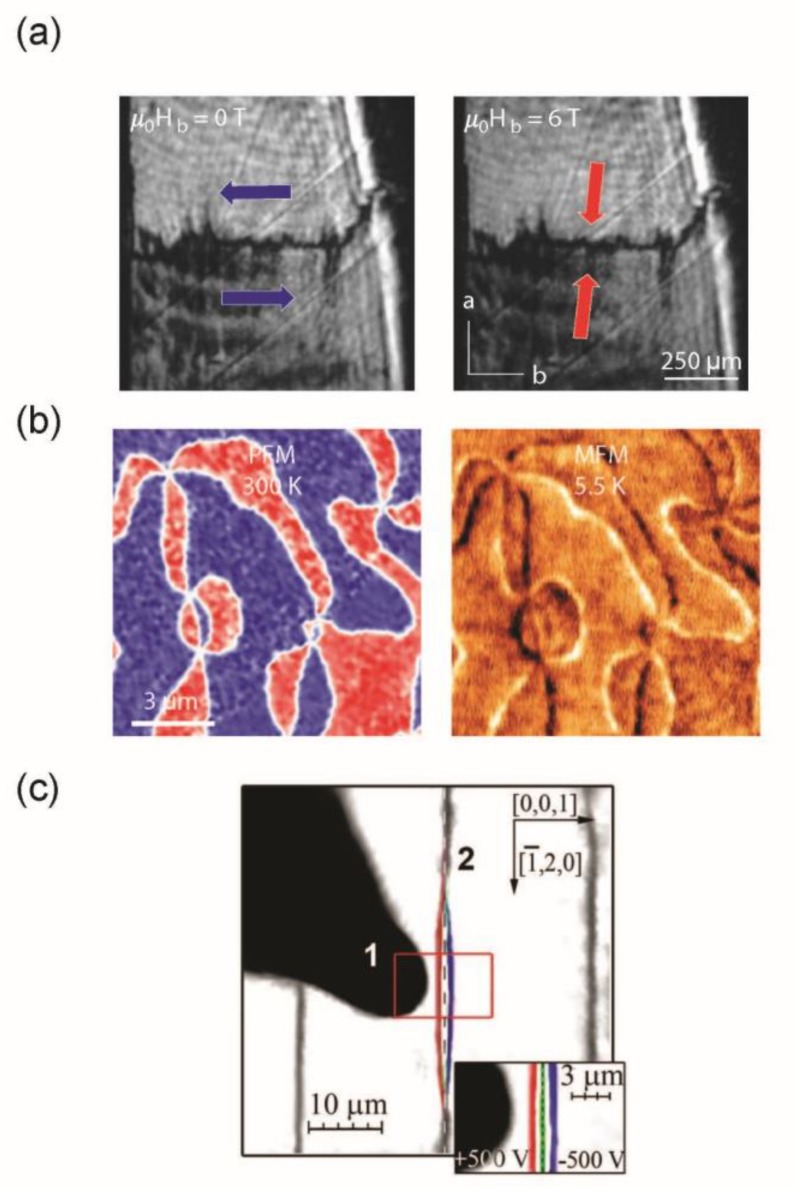
Spin-driven ferroelectric domain wall manipulation (**a**): Spatially resolved SHG images at 0 and 6 T show the magnetic field-induced 90° polarization flop in doped MnWO_4_ without a domain pattern change leading to nominally charged domain walls. The blue and red arrows in each image indicate the polarization direction [143]. Magnetic moment at antiferromagnetic/ferroelectric domain walls (**b**): Room temperature PFM and low-temperature MFM (5.5 K, 0.1 T) measurements on h-ErMnO_3_ [148]. Finite polarization at ferromagnetic Neel domains (**c**): Magneto-optical images of domain walls (2) in iron garnet films influenced by a voltage applied to a tip electrode; (1). Depending on the sign of the voltage, the domain wall is either attracted (red marked domain wall position) or repelled (blue marked domain wall position) from the electrode (3). The green line indicates the original position of the domain wall [149].

A direct clamping between both ferroic orders is found in type II multiferroics [137], where they appear jointly. A subgroup of type II multiferroics are the so-called spin-driven ferroelectrics. In a spin-driven ferroelectric material, the polarization is induced by an inhomogeneous magnetization pattern that breaks the inversion symmetry (antisymmetric magnetic exchange interaction, inverse Dzyaloshinskii–Moriya interaction, exchange striction mechanism, spin-dependent p/d hybridization) [140,141]. In these systems, the polarization can be manipulated with a magnetic field from controlling its polarization direction to a simple 90° flop or a full inversion. Optical techniques, notably SHG, have been used to resolve ferroic domain structures. In o-TbMnO_3_ [142] or MnWO_4_ [143], which normally show side-by-side (neutral) ferroelectric domain walls in zero magnetic field, an applied magnetic field flops the polarization by 90° without changing the domain structure (see Figure 3a). This leads to head-to-head and tail-to-tail domain configurations. Such a domain wall should be charged; however, direct measurements still need to be shown. In o-DyMnO_3_, charged domain walls can already exist close to the flop transition [144,145]. With such configurations, a direct on- and off-switching of conducting channels linked to the domain walls could be achieved. Another group of spin-driven ferroelectrics with a complex domain configuration are the rare-earth orthoferrites *R*FeO_3_ [146,147]. Because of the coupling between their electric and magnetic order parameters, a multitude of multiferroic domain walls can exist in these materials that can be purposely manipulated.

A drawback for most of these multiferroic materials, however, is that the ferroelectricity occurs together with antiferromagnetic ordering. An antiferromagnetic ordering shows no net magnetization and is, thus, difficult to access. However, recent studies have suggested that domain walls in multiferroic materials can possess a finite magnetic component. Magnetic force microscopy (MFM) studies under the application of a small magnetic field have revealed a magnetic moment at ferroelectric/antiferromagnetic domain walls in h-ErMnO_3_ [148]. Figure 3b shows the magnetic signal around a ferroelectric vortex core in h-ErMnO_3_ with the typical six-folded pattern. In BFO, theoretical calculations have shown that certain types of ferroelectric domain walls can have a finite magnetic moment [9,42,150,151] that is of the order of the remnant magnetization measured in BFO thin films [152]. Ferroelectric domain walls in BFO thin films have also been shown to exhibit magnetoresistance [153,154,155] indicating a more complex magnetic structure. In o-TbMnO_3_ thin films, experiments found a correlation between the number of ferroelastic domain walls and the magnetic moment of the material, suggesting that the domain walls possess a finite magnetic component [17,156].

The appearance of a finite magnetic moment at different domain walls in these ferroelectric/antiferromagnetic multiferroics suggests an unexpected complexity of magnetic structures with domain walls that have the possibility to be manipulated by electric and magnetic fields.

So far, we discussed materials where the magnetoelectric coupling at the domain walls is induced by their multiferroic domain properties. Unfortunately, magnetoelectric multiferroics mostly reside below room temperature, with rare exceptions like BFO. However, a wide range of ferroelectric and ferromagnetic materials do exist at room temperature. Recent experiments have shown that Neel-like domain walls in iron garnet films can possess a local polarization [149,157]. These localized multiferroic states are induced by an inhomogeneous magnetization pattern breaking the inversion symmetry (similar to the polarization in spin-driven ferroelectrics) [158] and exhibit a giant magnetoelectric effect. Such domain walls can be moved and transformed under electric field gradients [149,150,151,152,153,154,155,156,157,158,159,160] as well as exhibit electric modulations under AC magnetic fields [161]. Figure 3c shows a biased tip electrode close to a ferromagnetic domain wall in an iron garnet film. Depending on the polarization of the tip, the wall is either attracted or repelled, which is attributed to a ferroelectric component at the domain wall.

Inhomogenous magnetic patterns are not restricted to domain walls; they should also lead to an electric polarization at magnetic vortices and Neel-like skyrmions [158]. The feasibility of influencing skyrmions by an electric field has already been shown in multiferroic materials [162,163,164,165] and could extend to Neel-skyrmions in solely magnetic systems.

## 6. Solid-State Domain Wall Device Concepts

Novel functional properties of ferroic domain walls, as outlined in previous sections, have fueled conversations of their potential in nanoelectronic devices and interconnects. However, the practical realization of ferroelectric solid-state domain wall device concepts has remained elusive until recently. This has been primarily due to issues associated with the injection of ferroelectric domain walls at precisely defined spatial locations and their controlled long-range displacement. Additionally, problems associated with precise control of electrical conduction at domain walls, conventional device configurations, and lack of experiments with suitable prototype device geometries and compatible ferroelectric film orientations have plagued rapid developments in the field.

### 6.1. Ferroelectric Domain Wall Injection and Displacement

For a solid-state device comprising a ferroelectric film between two metal electrodes, the domain nucleation typically begins at one or more uncontrolled locations at the metal/ferroelectric interfaces. Therefore, the issue of the stochastic nature of domain nucleation and, as a result, the introduction of domain walls at random spatial locations must be addressed. Two strategies can be employed. The first one is based on tuning the strength of the applied field distribution in the ferroelectric film by introducing low permittivity structural defects (i.e., holes in the ferroelectric film) [166], such that regions with enhanced field strength—so-called hot-spots—can be created. These hotspots predetermine the spatial location of domain nucleation and, thus, domain walls. Further, by choosing the number and shape of these structural defects, multiple domain walls can be injected in a sequential manner at precisely defined spatial locations [167]. However, such an approach is hampered by the fixed nature of the domain wall injection sites. Besides, its applicability to thin films (< 100 nm) grown especially on single crystalline substrates remains an unexplored avenue. In the second approach, a low conductivity Pt top electrode on a ferroelectric film together with an electrically biased conductive scanning probe tip contacting the top electrode is employed [168,169]. These spatially defined domain wall injection sites are determined by the tip-top electrode contact point. The relatively high resistivity (10^−4^–10^−1^ Ωm) of the top electrode restricts the movement of the free charge in the electrode. As a result, the initial field (potential) distribution under the top electrode is not uniform. This leads to nucleation of a reverse domain and domain walls at the tip location. With time, the field distribution evolves to a uniform state causing switching under the entire electrode. The time-dependent electric field evolution thereafter drives the propagation of the initially injected domain walls. Using this approach, domain walls can be deterministically injected at arbitrary spatial locations and displaced controllably over several micrometers. By controlling the geometry of the top electrode, simple domain wall multiplier circuits working either in series or parallel were fabricated [168]. Additionally, domain wall velocities were tuned by over seven orders of magnitude by changing the thickness-dependent resistivity of electrodes and/or introducing local structural modifications in the electrodes [169]. The fastest domain wall speeds are around 0.1 ms^−1^, which is still approximately three to four orders of magnitude smaller than that needed for nanosecond operation of submicron devices. Further, the applicability of the tip-top electrode approach requires the conductivity of metals to be high enough to screen the polarization, but at the same time low enough to limit the charge transport. These requirements can be fulfilled (to some degree) for metal electrodes fabricated using electron-beam-induced deposition [169].

Another important aspect of ferroelectric domain wall displacement, which has been realized successfully, is the control over their direction of movement [2]. Typically, when an external field is applied, a pair of domain walls separated by opposite polarity domains move in opposite directions (i.e., towards each other or away from each other) depending upon the relative orientation of the applied electric field and polar domains. Thus, for a device that relies on wall motion, control over the direction of displacement of walls would be highly desirable. This crucial aspect can be realized by imposing a periodic sawtooth potential for the motion of domain walls. Such a potential profile can be introduced by sculpting a sawtooth morphology (thickness modulation) in the ferroelectric material, for instance, by selective focused ion beam (FIB) milling [170]. Unidirectional ferroelectric domain wall motion using this concept was experimentally demonstrated in single-crystal lamellas of KTiOPO_4_, and devices were termed as ferroelectric domain wall diodes. This proof-of-concept experiment is an important milestone and potentially a precursor for realizing more complex ferroelectric domain wall devices such as wall shift register and domain wall logic. Here, although ferroelectric domain wall device research is still in its initial stages, it can be based on analogous research in the more mature field of ferromagnetism, which has already delivered demonstrator devices (i.e., magnetic domain wall logic [171,172,173] and racetrack memories [174]).

### 6.2. Ferroelectric Domain Wall Memory

It took nearly a decade to realize a simple solid-state ferroelectric domain wall memory device after the initial discovery of domain wall conduction in ferroelectric oxides [30]. The simplest concept of a two-terminal solid-state domain wall device is one wherein a ferroelectric domain wall defines a path of low resistance for the transport of electrical charges and serves as an electrical interconnect between metal electrodes [3]. The device operation hinges on the presence (low resistance: ON state) or absence (high resistance: OFF state) of domain walls. However, there are practical difficulties with out-of-plane experiment geometries in which a ferroelectric is sandwiched between the top and bottom metal electrodes. This geometry precludes visualization of buried domain walls, direct probing of domain wall conductivity, and unambiguous assignment of wall conductivity as the principal mechanisms of device operation. These issues can be circumvented if one employs a coplanar geometry of electrodes along with a ferroelectric material with suitable crystalline orientation, for instance, fabrication of metal electrodes on the surface of (110)-oriented BFO thin films. The epitaxially grown (110)-oriented BFO thin films on SrTiO_3_ are special because these films display only two out of the eight possible polarization directions with their in-plane components parallel to the film surface (parallel to the morphological stripes; [1] direction). These two polarization directions can be switched and transformed into one another on the application of a coplanar electric field. Moreover, in the as-prepared (110) BFO, one polarization direction is much more dominant than the other. The (110) BFO, therefore, provides a clean slate for injection and removal of domains walls.

**Figure 4:**
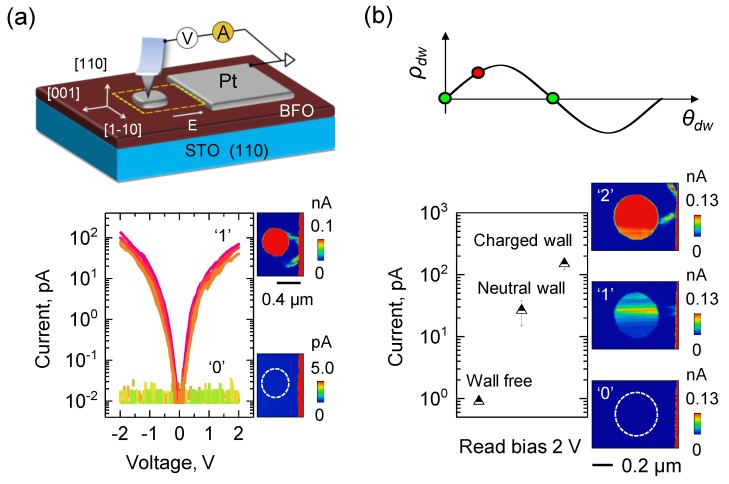
Domain wall memory and multilevel states: (**a**) Sketch of experimental geometry for a prototype domain wall memory device (top). *I-V* characteristics and current maps of the device in the presence (‘1′) and absence (‘0′) of domain walls (bottom). (**b**) Evolution of polarization charge, *ρ_dw_*, on domain wall plane versus wall angle, *θ* (top). Measured conduction levels and representative current maps of domain wall device for wall configurations of no wall, neutral, and charged walls (bottom) [3,175].

In such a prototype domain wall device, both the interelectrode spacing and the lateral dimensions of one of the electrodes are restricted to a few hundred nanometers (Figure 4a) to enable the relatively low-bias operation and precise injection of a domain wall pair between two metal electrodes [3]. The device exhibited relatively high OFF:ON ratios (~10^3^) with excellent endurance and retention characteristics. The encoding and nondestructive retrieval of information can be done at moderate biases, thus enabling low-energy operation. This study [3], therefore, provided direct experimental proof of a working nonvolatile solid-state domain wall device, wherein information is processed by ferroelectric domain walls.

### 6.3. Multilevel Domain Wall Devices

The domain wall device we discussed above displays binary states (i.e., an ON state and OFF state). However, multilevel states can also be realized in these devices. There are several routes to achieve multilevel states including sequential injection of conductive domain walls as well as tuning the domain wall length and charge. The sequential domain wall injection has been shown [167]; however, the experimental realization of a working device based on this concept is yet to be achieved. Recent developments towards device applications have, however, been made for tuning the domain wall length and its charge [3,175].

The resistance (*R*~ρ*d*; *ρ* and *d* are domain wall resistivity and length, respectively) of ferroelectric domain walls is directly proportional to the wall length. Therefore, domain walls of varying lengths can be thought of as the nanoscale analog of resistors of different resistances. As a result, one can potentially realize multilevel states in a two-terminal device by simply changing the wall length between metal electrodes. The wall length can, for instance, be influenced by the application of switching bias pulses of varying amplitude and duration. The feasibility of this idea was explored in an experiment in which domain wall devices with varying interelectrode spacings (from 400 to 90 nm), thus domain wall length, were prepared [3]. The noninvasive c-AFM maps reveal a stepwise change in the conduction state from a high-resistance state to a low-resistance state as a function of decreasing wall length. The conduction state shows an exponential dependence on wall length (*I*~e*^−d^*). Here, deep submicron interelectrode spacing (< 400 nm) is critical for the successful demonstration and operation of these domain wall devices. Further, the OFF:ON ratio (1 to ~10^3^) increases with decreasing lateral spacing (400 to 90 nm) of metal contacts and points to high scalability (i.e., sub-100 nm) of these devices [3]. 

Another pathway by which multilevel states can be achieved is through control of the charge state of domain walls (Figure 4b). Using electric fields, stable charged, neutral, or no domain wall states are selectively and alternatively injected or erased between two metallic electrodes giving reversible, precise, stable conformational control over the charge state of ferroelectric domain walls [175]. This is analogous to conformational switches in molecular electronics. The main factors that enable this conformational control for domain walls are (i) strength and/or temporal profile of the applied inhomogeneous electric field distribution between metal contacts (i.e., width/height of writing bias pulses), and (ii) electrons as majority carriers in BFO stabilizing the head-to-head domain walls. The device transitions from an OFF to a low-resistance ON state upon the injection of a neutral domain wall changing its conduction by more than an order of magnitude (Figure 4b). Thereafter, upon transition to a charged domain wall state, the resistance is lowered by another order of magnitude.

### 6.4. Nondestructive Readout of Ferroelectric Domains

In devices discussed thus far, the information is stored in domain walls. However, domain walls can also be employed to read information that is stored in the form of ferroelectric domains, as implemented in complementary metal–oxide–semiconductor (CMOS)-compatible commercialized ferroelectric random-access memories (FeRAM) [176,177]. In FeRAMs, the information is stored in the form of domains and is read through a destructive charge integration scheme. As a result, the information needs to be re-encoded after being read and, thus, increases energy consumption. Furthermore, the charge-based readout scheme severely limits scalability to below 200 nm [178]. In a recent study by Jiang et al. [179], they proposed an alternative new mechanism to read the encoded domains, which is nondestructive, and involves the creation of transient, highly conducting domain walls. The read operation is based on the measurement of electrical current rather than charge and, thus, is of the resistive type. The read protocol involves the application of a short bias pulse of amplitude higher than the coercive bias. Now, if the polar orientation of the encoded domain is opposite to that of the applied read field, partial switching occurs and leads to the formation of highly conducting, charged domain walls. Hence, a high read-out electrical current (state ‘1′) is measured with contributions from both the partial switched area and the conducting, uncompensated charged domain walls. The strong depolarization field from the uncompensated polarization charges drives the polarization back to its original state and leads to the disappearance of charged walls immediately after removal of the read pulse. For the opposite polarity domains (state ‘0′), because now the polarization is parallel to the applied read electric field, no switching will occur, and, as a result, no read-out current will be detected. Using this approach, memory cells employing mesa-like BFO with an in-plane electrode geometry were demonstrated [179]. The observed read-current for state ‘1′ of ~14 nA is sufficiently high to enable nanosecond (~10 ns) operation. Other experiments [180] even achieved read currents of up to ~300 nA by changing the relative orientation between the applied read field and polarization direction. In this scheme, sub-100 nm memory cells were realized, and the devices are presumably free of undesirable effects associated with the accumulation of mobile charged defects and/or oxygen vacancies at the walls. Both two-terminal and three-terminal device configurations [179] were achieved showing excellent endurance (10^7^–10^9^ cycles) and stability of operation. For two-terminal device configurations, the OFF:ON ratio was around 100, while for the three-terminal devices it was ~3.

### 6.5. Other Novel Device Concepts

Multiferroic tunnel junctions (MTJs) exploit the quantum phenomenon of tunneling electrons. In MTJs, the tunneling current between magnetic electrodes separated by an ultrathin ferroelectric barrier strongly depends upon the relative orientation of the magnetic moments of the electrodes and the polarization direction of the ferroelectric [181,182,183]. By selecting various combinations of polar and magnetic moment vectors, four states can be realized. In addition, continuously tunable multilevel states in ferroelectric tunnel junctions (FTJs) were also achieved because of fractional switching of polarization and/or hysteretic ionic effects. However, the role of domain walls in these devices did not seem important until recently. Sanchez-Santolino et al. [4] studied the impact of an isolated head-to-head domain wall confined to an MTJ comprising of ferromagnetic La_0.7_Sr_0.3_MnO_3_ (LSMO) electrodes separated by an ultrathin (4–5 nm) BaTiO_3_ layer. This head-to-head domain wall appears in the pristine state of the BaTiO_3_ and is caused by polarization pinning at the symmetric polar interfaces (TiO_2_/La_1−x_Sr_x_O) with an effective ionic charge of +0.35 e^−^. The polar disruption at the head-to-head wall was argued to be sustained by electron donor oxygen vacancies, and it confines electrons to a wedge-shaped quantum potential well. This confinement drives energy quantization of electronic states in the well and affects the electron tunneling current of the device. Experiments below 100 K reveal conductance oscillations in the measured tunneling current and point to resonant transport through discrete unoccupied electronic states confined to the wall [4,184]. The measured energy gap between almost equally spaced discrete levels was around 70–90 meV. Quantum transport calculations [184] further found resonant tunneling through confined electronic states as an orbital selective process and suggest the use of domain walls embedded in MTJs as an alternative avenue to affect electronic transport at the nanoscale.

Domain walls have recently also made an impact in the field of tunable dielectrics. Most telecommunication devices rely on the tunability of their frequency or the frequency agility in the radio spectrum. The usefulness of ferroelectrics in this area is well established [185]. However, domain walls were considered detrimental to the performance of these devices due to high dielectric losses and hysteretic device response under an applied electric field [186]. To avoid these effects, ferroelectric materials are typically operated above their Curie temperature T_C_ [187], where the dielectric tunability is rather limited. Recent work by Gu et al. [188] challenges conventional wisdom on domain walls and shows that these boundaries can be positively exploited to achieve high performance. In their study, dielectric responses of strained Ba_x_Sr_1−x_TiO_3_ (BST) films with a carefully chosen composition (i.e., x = 0.8) were investigated. The domain wall phase diagram of BST exhibits a vertex at room temperature and, thus, a high variety of domain walls (domain size ~100 nm) in the as-prepared state. The experimental results showed gigahertz microwave tunability (1–10 GHz) and low dielectric loss. The device performance was comparable to that of bulk single crystals but was superior by a few orders of magnitude to state-of-art thin-film devices. Resonant domain wall fluctuations or oscillations in a domain wall rich sample, owing to proximity and accessibility of different domain wall variations, were determined as the main factors behind the observation of low dielectric losses and the exceptionally high-quality factors [188].

Another phenomenon that could potentially benefit from properties of ferroic domain walls is the negative capacitance in ferroelectrics. Ferroelectric materials with negative capacitance effects [189,190,191] have been proposed to provide a way forward for further miniaturization and lower power consumption for the field effect transistor basic building blocks of CMOS technology. The negative capacitance effect was first detected in experimental studies on ferroelectric capacitors [192,193,194,195,196]. In a study by Zubko et al. [197], stable negative capacitance was demonstrated for multidomain ferroelectric–dielectric superlattices across a wide range of temperatures. Using phenomenological modeling, wall motion was shown to give rise to negative permittivity and enhance the temperature range over which the negative capacitance can be observed. Alternatively, a nanosized ferroelectric domain nucleus has been proposed to exhibit a static, stable negative capacitance effect [198].

## 7. Summary

Domain walls and their unique properties continue to be widely explored. Over recent years researchers have gained significant new insight into fundamental properties and potential applications. Of special note is the utilization of the intrinsic structure of walls and the control of wall properties through order parameter coupling in multiferroic systems [25]. Furthermore, light interaction with domain walls and associated control of their properties opens new research applications of domain walls in optoelectronics, which will likely see strong development in the coming years.

Looking at the evolution of nanoelectronics devices based on domain walls, it is especially interesting to see how far the negative capacitance concept can be developed and if it can be applied in commercial devices [189,190,191]. Regarding domain wall memory, so far, simple two-terminal binary and multilevel domain wall devices show promising characteristics [3,175], although the question of whether they will be any better than (or can even compete with) existing technologies remains and needs to be addressed further. The main factors that can potentially determine this, but are yet to be critically explored, include the speed of operation, retention, energy consumption, and integration with existing semiconductor architectures [199]. Nevertheless, research on domain walls has seen considerable progress and interest over the last few years and lays the foundation for future research in this direction.
